# Assessing the Outcome of Adult Kidney Transplantation from a Deceased Expanded Criteria Donor: A Descriptive Study

**DOI:** 10.7759/cureus.11199

**Published:** 2020-10-27

**Authors:** Mona Alshahrani, Mutlaq Alotaibi, Burhan Bhutto

**Affiliations:** 1 Nephrology, McMaster University, Hamilton, CAN

**Keywords:** outcome, expanded criteria donor, transplantation, kidney donor profile index

## Abstract

Background

End-stage renal disease (ESRD) creates a great burden on the quality of life. Patients after kidney transplantation have been reported to have a greater quality of life and better outcomes health outcomes. Therefore, it is important to optimize the best method of following well-constructed criteria such as the expanded criteria donor (ECD) to reduce the chances of rejection rate and deaths post-transplantation particularly in elderly patients in conjunction with the kidney profile donor index (KDPI).

Methods

This is a retrospective descriptive study of all patients who received kidney transplantation from a deceased donor from the ECD as well as ECD with donation after cardiac death (DCD) at St. Joseph Health Care Hospital over a 24 month time period from January 2017 to January 2019. All adult recipients from standard criteria donor (SCD) and living donors were excluded from the study.

Results

The study included 60 patients with 36 (60%) from the ECD and 24 (40%) were from the ECD/DCD group. The most common cause of ESRD among recipients was diabetes mellitus (DM) involving 23 (38.3%) of the patients. The creatinine outcome was the highest in the ECD/DCD group at one month (211 ± 71) and the lowest creatinine recorded was also in the ECD/DCD at 12 months (160 ± 78). Lastly, only four patients died in 12 months and only six recipients reported graft loss over 12 months.

Conclusion

Descriptive data of the included ECD/DCD showed increase trend in survivability of the recipients when used among the elderly, giving us more insight on the benefits of ECD/DCD transplantation.

## Introduction

Kidney transplantation proves to be the best treatment of choice for most patients with end-stage renal disease (ESRD). Generally, survival and quality of life are found to be superior in allograft recipients compared with patients on the deceased donor waitlist [1]. This benefit is also observed among recipients over the age of 60 years with a relatively beneficial noticeable reduced risk of cardiovascular events [2,3]. Moreover, there is a large gap between the number of patients waiting for a transplant and the number receiving a transplant, which leads to a renewed interest in the use of expanded criteria donor (ECD) kidneys to increase the donor pool particularly for the elderly recipients [4,5].

The Organ Procurement and Transplantation Network (OPTN) instituted a formalized definition of marginal kidneys in 2002 with the advent of ECD [5]. According to Metzger et al., ECD kidneys are those either from a deceased donor ≥ 60 years of age, or a donor 50 to 59 years of age with at least two of the following features: history of hypertension, terminal serum creatinine > 1.5 mg/dL (133 mmol/L), or cerebrovascular cause of death [5]. Such criteria were defined based on the presence of variables that had an increased risk of graft failure by 70% in comparison with the standard criteria donor kidney (SCD) [5]. Allocation policies from the United Network for Organ Sharing (UNOS) require those who are on the transplantation waiting list to be given the choice to be included in the ECD kidneys and those who are included in that list are eligible to receive SCD kidneys as well [6].

Studies suggest that kidneys from donors over the age of 60 may have suboptimal graft survival, with a three-year graft survival being only 58% [7] while a recent study suggests that the new age of worsening survival rate has increased to 70 years of age in the same group [8]. Therefore, the donor age is thought to be the most significant factor that can negatively impact graft survival [9]. The kidney profile donor index (KDPI) score has been used to assess the risk of graft loss of any given donor compared to the median population of donors using 10 donor-specific criteria including age, sex, height, weight, history, and duration of hypertension and diabetes, hepatitis C virus (HCV) status, donation after cardiac death (DCD) status, and donor cause of death. Therefore, the KDPI index has been used to identify organs that may have decreased survival compared to the SCD groups, and scores of >=85 are thought to be associated with poorer long-term allograft survival [10]. A donor kidney with a KDPI score of ≥85 has been characterized in the literature as having similar donor characteristics as an ECD kidney [11]. Traditionally, donor kidneys that were classified as ECD have been shown to have decreased allograft survival compared to SCD kidneys. By 2006, most Canadian organ procurement organizations, including Ontario’s Trillium Gift of Life Network (TGLN) started using the ECD classification to allocate deceased donor kidneys. Despite the increasing use of ECD kidneys for transplantation, the outcomes of kidneys from deceased donors with ECD characteristics has received little attention in literature. For this reason, we conducted this study to assess the kidney transplant outcome from ECD donors at St. Joseph Health Care Hamilton, Ontario, Canada, and is the first study to be conducted in our center.

## Materials and methods

Patients

We included in this retrospective descriptive study all adult renal transplant recipients who received a deceased donor kidney from an ECD from January 2017 to January 2019 at St. Joseph Health Hamilton, Ontario in Canada. We excluded the living kidney transplant recipients, non-ECD donors, and KDPI that cannot be calculated based on the donor information available. The study protocol was approved by the Hamilton Integrated Research Ethics Board. Data was collected from Ontario’s Trillium Gift of Life Network (TGLN) donor charts and recipients’ charts in the St. Joseph Health Care System using Epic (Epic Systems Corporation, Verona, WI, USA).

Study population

A total of 60 adult recipients (age ≥ 18 years) who received an ECD kidney transplant from January 2017 to January 2019 were eligible for the study including those with a prior kidney transplant and ECD/DCD donors. Adult recipients from SCD and living donors were excluded from the study.

Outcomes

The primary outcomes were renal function at 12 months measured by serum creatinine, total graft loss defined as the time from transplantation to a composite of return to chronic dialysis, pre-emptive re-transplant or death with graft function, all-causes of mortality, and primary nonfunction graft which was defined as a failed function of the transplanted kidney that necessitated continued maintenance dialysis. The secondary outcomes were incidence of delay graft function (DGF) defined as kidney function that ultimately supported the patient but necessitated post-transplantation dialysis within seven days after kidney transplant, biopsy-proven allograft rejection, the incidence of cytomegalovirus (CMV), and BK virus (BKV) in the first 12 months after the kidney transplant and length of stay in the hospital after the transplant.

## Results

Out of the 60 patients, 36 (60%) belonged to the ECD while 24 (40%) belonged to the ECD and DCD group. Regarding age, the mean age of the recipient group was 68 ± 7.6 years while the mean age of the donor was 62 ± 4.8 years. In addition, the mean body mass index (BMI) of the recipients and donors was 28 ± 6 years and 29.4 ± 6.8 years, respectively. As well, 23 recipients (38.3%) had diabetes mellitus (DM) as the cause of ESRD making it the most common cause, followed by glomerulonephritis with 15 reported cause (25%), and the least common cause was polycystic kidney disease in three patients (5%). Most recipients had hemodialysis at baseline therapy 43 (71.7%) compared to only 15 (25%) towards peritoneal dialysis and two (3.3%) patients with a failed kidney transplantation. Regarding the choice of an induction agent, 46 (76.6%) patients received basiliximab alone, 11 (18.3%) recipients received anti-thymocyte globulin (ATG), and only 3 (5%) received both (Table 1).

**Table 1:** Baseline Recipient Characteristics BMI: Body Mass Index, cPRA: Calculated Panel Reactive Antibody, ECD: Expanded Criteria of Donor, DCD: Donation after Cardiac Death, ESRD: End-Stage Renal Disease, HLA: Human Leukocyte Antigen, n: Numbers, SD: Standard Deviation.

Baseline Recipient characteristics										
		All (n = 60)			ECD (n = 36)				ECD and DCD (n = 24)	
		n	%		n			%	n	%
Numbers		60	100%		36			60	24	40
Age mean ± SD		68 ± 7.6			68 ± 8.7				68.5 ± 5.6	
BMI mean ± SD		28 ± 6			27 ± 6				27.8 ± 6	
Sex	Male	40	66.7%		22			61 %	18	75%
	Female	20	33.3%		14			39%	6	25%
Cause of ESRD	Diabetes mellitus	23		38.3%	12		33.3%		11	45%
	Hypertension/Reno-vascular Disease	8		13.3%	7		19.4%		1	4.2
	Polycystic Kidney Disease	3		5%	1		2.8%		2	8.3%
	Glomerulonephritis	15		25%	7		19.4%		8	33.3%
	Other	10		16.6%	8		22.2%		2	8.3%
	Unknown	1		1.7%	1		2.8%		0	0%
Renal replacement therapy at baseline	Failed Kidney Transplant	2		3.3%	2	5.6%			0	0%
	Hemodialysis	43		71.7%	26	72.2%			17	71%
	Peritoneal Dialysis	15		25%	8	22.2%			7	29%
Number of transplant	First	56		93.3%	32		88.9%		24	100%
	Second	4		6.7%	4		11.1%		0	0%
Induction	Basilixmab	46		76.7%	28		77.8%		18	75%
	Anti-thymocyte globulin	11		18.3%	8		22.2%		3	12.5%
	Both	3		5%	0		0%		3	12.5%
HLA-DR Match	0 Match	40		66.7%	24		64.9%		16	66.7%
	1 Match	17		28.3%	10		27%		7	29.2%
	2 Match	3		5%	2		5.4%		1	4.2%
	Unknown	1		1.7%	1		2.7%		0	0%
HLA-DQ Match	0 Match	28		46.7%	17		45.9%		11	44%
	1 Match	27		45%	16		43.2%		11	44%
	2 Match	6		10%	3		8.1%		3	12%
	Unknown	1		1.7%	1		2.7%		0	0%
Warm ischemia (Minutes)	Mean	38 Minute			38 Minute				38 Minute	
	SD	± 7.4			± 8				± 6	
	Minimum	26 Minute			27 Minute				26 Minute	
	Maximum	57 Minute			57 Minute				50 Minute	
cPRA	Mean	21%			25%				15%	
	SD	± 31			± 34				± 22	
	Minimum	0%			0%				0%	
	Maximum	100%			100%				73%	
HLA mismatch	Mean	11			11				11	
	SD	± 3.2			± 3				± 3	
	Minimum	0			0				2	
	Maximum	17			17				15	

Donors were mostly of white ethnicity 51 (85%) with most of them in the ECD and DCD group 23 (95.8%). Meanwhile, 28 individuals from the donor group were hypertensive compared to only six diabetics. Regarding the cause of death in the donor groups, cerebrovascular accidents were the most dominant with 38 donors (63.3%) followed by head trauma 8 (13.3%), anoxia 6 (10%), other 6 (10%) and central nervous system tumor 2 (3.3%) (Table 2). The overall mean warm ischemia time in the ECD was 38 ± 8 minutes, and the time in the ECD and DCD was almost similar at 38 ± 6 minutes. The human leukocyte antigen (HLA)-mismatch was identical in both groups with a mean value of 11± 3. In addition, HLA-DR showed the least match with 0 match in 40 recipients (66.7%) and only 17 patients had one match (28.3%), and the HLA-DQ was distributed almost evenly between a 0 match and one match with 28 (46.7%) and 27 (45%), respectively. Lastly, the mean value of KDPI in the donor ECD group was 82 ± 10.5 and the mean value of the KDPI in the donor ECD and DCD group was 82 ± 7 (Table 2).

**Table 2:** Baseline Donor Characteristics Cr: Creatinine, DCD: Donation after Cardiac Death, ECD: Expanded Criteria of Donor, HCV: Hepatitis C Virus, KDPI: Kidney Donor Profile Index, n: Numbers, SD: Standard Deviation

Baseline Donor Characteristics							
		All		ECD		ECD and DCD	
Numbers		n	%	n	%	n	%
		60	100%	36	60	24	40
Age mean ± SD		62 ± 4.8		62 ± 5.7		62.5 ± 3.4	
BMI mean ± SD		29.4 ± 6.8		29 ± 6.9		29.9 ± 6.9	
Ethnicity	White	51	85%	28	77.8%	23	95.8%
	Asian	6	10%	5	13.9%	1	4.2%
	Hispanic	2	3.3%	2	5.6%	0	0%
	African American	1	1.7%	1	2.8%	0	0%
	Others	0	0%	0	0%	0	0%
Hypertension	0-5 Y	12	20%	4	11.1%	11	37.5%
	6-10 Y	4	6.7%	4	11.1%	0	0%
	> 10 Y	2	3.3%	1	2.8%	1	4.2%
	Unknown Duration	10	16.7%	8	22.2%	2	8.3%
Diabetes Mellitus	0-5 Y	3	5%	1	2.8%	2	8.4%
	6-10 Y	2	3.3%	0	0%	2	8.4%
	> 10 Y	1	1.7%	1	2.8%	0	0%
	Unknown	0	0%	0	0%	0	0%
Cause of Death	Cerebrovascular Accident	38	63.3%	22	61.1%	16	66.7%
	Head Trauma	8	13.3%	5	13.9%	3	12.5%
	Anoxia	6	10%	3	8.3%	3	12.5%
	Central Nervous System Tumor	2	3.3%	2	5.6%	0	0%
	Other	6	10%	4	11.2%	2	8.4%
HCV Status	Negative	57	95%	34	94.4%	23	95.8%
	Positive	3	5%	2	5.6%	1	4.2%
Serum Cr (mmol/l)	Mean	68.7		73.3		61.9	
	SD	± 24.1		± 26.6		± 18.11	
	Minimum	33		33		34	
	Maximum	151		151		105	
KDPI	Mean	82		82		86	
	SD	± 10		± 10.5		± 7	
	Minimum	59		59		72	
	Maximum	97		97		97	

Primary outcome

The mean creatinine value of one month was 183 ± 112 in all groups and was the highest among ECD and DCD with a mean value of 211 ± 71, and it was lower in the ECD group with a mean value of 165 ± 131. The creatinine value of six months showed a decrease in the ECD and DCD group reaching 178 ± 91 while it slightly decreased in the ECD group to 171 ± 117. Lastly, the creatinine 12 months value in the ECD and DCD decreased further to reach a mean value of 160 ± 78. However, in the ECD group, the creatinine 12-month value increased to 185 ± 206. Furthermore, the urine protein value showed an overall steady decrease in both groups with one-month, six-month, and 12-month mean values reported as 32 ± 74, 16 ± 35, and 11 ± 22, respectively (Table 3). As well, the overall graft loss at 12 months in both groups was 6 (10%) with four from the ECD group and two from the ECD and DCD group. Moreover, only two deaths occurred at the 12 months mark with one death from each group.

**Table 3:** Outcomes AMBR: Antibody Mediated Rejection, BPAR: Biopsy-Proven Acute Rejection, CMV: Cytomegalovirus, cPRA: Calculated Panel Reactive Antibody, Cr: Creatinine, DCD: Donation after Cardiac Death, DSA: Donor Specific Antibody, ECD: Expanded Criteria of Donor, ESRD: End-Stage Renal Disease, HLA: Human Leukocyte Antigen, LOS: Length of Stay, n: Numbers, SD: Standard Deviation, TCMR: T-Cell Mediated Rejection.

Primary Outcome													
				All (n = 60)				ECD (n = 36)			ECD and DCD (n = 24)		
Cr 1-month (umo/l) Mean ± SD				183 ± 112				165 ± 131			211 ± 71		
Cr 6-month (umo/l) Mean ± SD				174 ± 107				171 ± 117			178 ± 91		
Cr 12-month (umo/l) Mean ± SD				175 ± 167				185 ± 206			160 ± 78		
Urine protein 1-month mg/mmol Mean ± SD				32 ± 74				35 ± 89			12 ± 0.7		
Urine protein 6-month mg/mmol Mean ± SD				16 ± 35				15 ± 33			18 ± 39		
Urine protein 12-month mg/mmol Mean ± SD				11 ± 22				10 ± 18			2.7 ± 28		
LOS Mean ± SD				10 ± 5				16 ± 7			9.5 ± 4		
				n		%		n		%	n		%
Graft loss 12 month				6		10%		4		11%	2		8.3%
Patient death 12 month				2		3.3%		1		2.8%	1		4.2%
Primary non function allograft				3		5%		3		8.3%	0		0%
Secondary Outcome													
				All				ECD			ECD and DCD		
				n		%		n		%	n		%
Delayed Graft Function				20		33.3%		9		25%	11		45.8%
CMV Viremia				19		32%		11		30.6%	8		33.3%
BK Viremia				17		28.3%		7		19.4%	10		41.7%
Rejection in 12 months				4		6.7%		3		8.3%	2		8.3%
Antibody Mediated Rejection				1		1.6%		1		2.8%	0		0%
T-Cell-Mediated Rejection				3		75%		1		2.8%	2		8.3%
Number of death in 12/months Post-Transplant: 2 patients 3.3%													
	Age/Gender	Cause of ESRD	Time on Dialysis		Date of Transplant		Date of Death		Time Post-Transplant			Cause of Death	
N1	79/M	Focal Segmental Glomerulosclerosis without Diabetes	4 years		16/09/2018		03/02/2019		4 Months			Small Bowel Obstruction /Sepsis	
N2	74/M	Diabetic Nephropathy	5 years		22/12/2017		04/02/2018		2 Months			Sepsis/Perforated bowel.	
Rejection Rate: 4 patients 6.7 %													
#	Date of Treatment	Date of BPAR	Type of Rejection		cPRA		HLA Mismatch		Type of Induction			Evidence of non-adherence	
1	21/01/2019	03/07/2019	Borderline TCMR		30%		13/18 0DR 1DQ		Thymoglobulin			No, severe cmv viremia, refuse treatment, lost the graft	
2	09/06/2018	14/06/2019	ABMR no DSA		78%		11/18 0DQ 0DR		Thymoglobulin			No, treated Cr 200s	
3	29/01/2017	15/11/2017	Borderline TCMR		0		11/18 1DR 1DQ		Basiliximab			No, CMV pos, medications reduced, Cr 120	
4	17/12/2017	28/09/2018	Borderline TCMR		0		7/18 1DQ 1DR		Basiliximab			No, Tac level 4.4-5.5 BK pos, treated Cr 200s	

Secondary outcome

Regarding a secondary outcome, the overall numbers of patients with a delayed graft function were 20 (33%), nine from the ECD, and 11 from the ECD and DCD group. When comparing CMV and BK viremia, they were almost similar with 19 (32%) individuals infected with CMV and 17 (28.3%) reported BK viremia. However, CMV viremia was higher in the ECD group with 11 recorded infections compared to 10 BK viremia cases in the ECD and DCD group. Regarding transplant rejection outcome, only four recipients were reported to have rejection at the 12th month with only one reported antibody-mediated rejection in the ECD, and three had T-cell-mediated rejection (Table 3).

The first reported death at the 12-month period was that of a 79-year-old male who had been on dialysis for four years due to ESRD caused by focal segmental glomerulosclerosis (FSGS) without diabetes. The patients survived four months after his transplant and died due to small bowel obstruction accompanied by sepsis. The second reported case was a 74-year-old male with diabetic neuropathy as the culprit of ESRD. Similarly, he had been on dialysis for five years and passed away two months after the transplant due to small bowel perforation and sepsis (Table 3).

As previously stated, four recipients reported a rejection pattern of 12 months without any reports of non-adherence. The first case was an antibody-mediated rejection type with no donor specific antibody. The patient’s calculated panel reactive antibody (cPRA) was 78% and the HLA mismatch was reported as 11/18 0DQ 0DR, and the induction agent of choice was thymoglobulin. The second case, however, was similar except that the rejection pattern was T-cell mediated rejection with cPRA of 30% and an HLA mismatch of 13/18 0DR 1DQ. Also, the last two cases had basiliximab as an induction agent with no reported cPRA 0% and a borderline T-cell mediated rejection (Table 3).

Lastly, death censored graft loss was immediate in three cases and was delayed in the fourth one 10 months after the transplant. The first case was a 69-year-old patient with ESRD caused by urinary tract infection and had a delayed rejection that occurred after 10 months. The main cause of the allograft loss was recurrent CMV viremia and the donor was 69 years old from the ECD group with a KDPI of 86%. The second case was a 78-year-old patient with ESRD caused by hypertension with an immediate rejection of the graft. The main cause of the allograft loss was a primary nonfunctional cause where the donor was 67 years old from the ECD and DCD group with a KDPI of 89% and was severely atherosclerotic. The third case was that of a 74-year-old patient with FSGS as the cause of ESRD and immediate rejection. The donor was 66 years old from the ECD group with a KDPI of 95%. Lastly, the final case of a 47-year-old patient with ESRD caused by FSGS who also had an immediate rejection and the donor was 71 years old from the ECD group with a KDPI of 91% (Table 4).

**Table 4:** Death Censored Graft Loss CMV: Cytomegalovirus, DCD: Donation after Cardiac Death, ECD: Expanded Criteria of Donor, ERSD: End-Stage Renal Disease, FSGS: Focal Segmental Glomerulosclerosis, HTN: Hypertension, KDPI: Kidney Donor Profile Index, TCMR: T-Cell Mediated Rejection, TMA: Thrombotic Microangiopathy, UTIs: Urinary Tract Infections.

	Recipient Age	Cause Of ESRD	Time from Transplant	Cause of Allograft Loss	Donor Age	Type of Donor	KDPI
Case 1	69 Y	UTIs	10 month	Recurrent CMV viremia, TCMR and immunosuppression intolerance	64 Y	ECD	86%
Case 2	78 Y	HTN	Immediate	Primary non function (severe atherosclerotic donor kidney)	67 Y	ECD/DCD	89%
Case 3	74 Y	FSGS	Immediate	Primary non function (TMA)	66 Y	ECD	95%
Case 4	42 Y	FSGS	Immediate	Primary non function (recurrent FSGS)	71 Y	ECD	91%

## Discussion

The growing gap between demand and supply for kidney transplants leads to renewed interest in the use of ECD kidneys to increase the donor pool. In Canada, kidneys from deceased donors makes up approximately 60% of the donor pool over the last decade [12]. Although most studies of ECD kidney transplantation confirm lower allograft survival rates and, generally, worse outcomes than SCD kidneys, recipients of ECD kidneys generally have improved survivability in comparison to the wait-listed dialysis patients, thus encouraging the pursuit of this type of kidney transplantation [5]. Our study is the first study that considers the outcome of kidney transplantation using the ECD kidneys at St. Joseph Hospital in Hamilton. Our results show a favorable outcome comparable with Canadian outcomes [13].

According to Young et al., total graft loss was 10% vs 11.4% in the first-year post transplantations [13]. In comparison, death censored graft loss is 6.9% vs 6.6% in our data. According to Wu WK et al., the risk of DGF was more likely to occur in male and older donors and is similar to our findings where all rejections occur from donors above the age of 70 years [14]. Descriptively, the survival rate in the first year post-transplantation patients is 97% when compared to the literature of the United States and Canada with >90% survival rate [15,16]. So, it appears that the utilization of extended criteria donor kidneys can significantly improve survival for older recipients, and we should encourage using these ECD kidneys for the appropriate matching ages. The limitations of our study were a small patient sample and short term follow up. We did not include the outcome of kidney transplantation using SCD in our center as a comparable group. Furthermore, the data was not statistically analyzed to do so once a larger sample size was collected and followed up over the coming years. Our future direction is to follow-up with the recipients over a three- to five-year period and compare the survival and graft function to SCD donors at our center. Moreover, we will calculate the cold ischemic time, and donor KDPI and correlate it with the actual recipient outcome.

## Conclusions

Descriptive data of renal transplantation recipients with ECD kidneys at St. Joseph Hospital resulted in initial favorable outcomes when compared with other programs in Canada as well as global programs. The use of these organs should continue to be more widely considered given the high morbidity and mortality experienced by patients on dialysis. 
